# Peretinoin, an acyclic retinoid, suppresses steatohepatitis and tumorigenesis by activating autophagy in mice fed an atherogenic high-fat diet

**DOI:** 10.18632/oncotarget.18116

**Published:** 2017-05-23

**Authors:** Hikari Okada, Riuta Takabatake, Masao Honda, Kai Takegoshi, Taro Yamashita, Mikiko Nakamura, Takayoshi Shirasaki, Yoshio Sakai, Tetsuro Shimakami, Naoto Nagata, Toshinari Takamura, Takuji Tanaka, Shuichi Kaneko

**Affiliations:** ^1^ Department of Gastroenterology, Kanazawa University Graduate School of Medicine, Kanazawa, Japan; ^2^ Department of Advanced Medical Technology, Kanazawa University Graduate School of Health Medicine, Kanazawa, Japan; ^3^ Department of Cell Metabolism and Nutrition, Brain/Liver Interface Medicine Research Center, Kanazawa University, Kanazawa, Japan; ^4^ Department of Disease Control and Homeostasis, Kanazawa University Graduate School of Medical Science, Kanazawa, Japan; ^5^ The Tohkai Cytopathology Institute, Cancer Research and Prevention, Gifu, Japan

**Keywords:** acyclic retinoid, non-alcoholic fatty liver disease (NAFLD), hepatocellular carcinoma (HCC), autophagy

## Abstract

The pathogenesis of non-alcoholic steatohepatitis (NASH) is still unclear and the prevention of the development of hepatocellular carcinoma (HCC) has not been established. We established an atherogenic and high-fat diet mouse model that develops hepatic steatosis, inflammation, fibrosis, and liver tumors at a high frequency. Using two NASH-HCC mouse models, we showed that peretinoin, an acyclic retinoid, significantly improved liver histology and reduced the incidence of liver tumors. Interestingly, we found that peretinoin induced autophagy in the liver of mice, which was characterized by the increased co-localized expression of microtubule-associated protein light chain 3B-II and lysosome-associated membrane protein 2, and increased autophagosome formation and autophagy flux in the liver. These findings were confirmed using primary mouse hepatocytes. Among representative autophagy pathways, the autophagy related (Atg) 5-Atg12-Atg16L1 pathway was impaired; especially, Atg16L1 was repressed at both the mRNA and protein level. Decreased Atg16L1 mRNA expression was also found in the liver of patients with NASH according to disease progression. Promoter analysis revealed that peretinoin activated the promoter of Atg16L1 by increasing the expression of CCAAT/enhancer-binding-protein-alpha. Interestingly, Atg16L1 overexpression in HepG2 cells inhibited palmitate-induced NF-kB activation and interleukin-6-induced STAT3 activation. We showed that Atg16L1 induced the de-phosphorylation of Gp130, a receptor subunit of interleukin-6 family cytokines, which subsequently repressed phosphorylated-STAT3 (Tyr705) levels, and this process might be independent of autophagy function. Thus, peretinoin prevents the progression of NASH and the development of HCC through activating the autophagy pathway by increased Atg16L1 expression, which is an essential regulator of autophagy and anti-inflammatory proteins.

## INTRODUCTION

Hepatocellular carcinoma (HCC) is one of the most common malignancies with a particularly poor outcome. The recent increase in non-alcoholic fatty liver disease (NAFLD) associated with metabolic syndrome could be a strong risk factor for HCC. [1] In Japan, non-B and non-C HCC, which is negative for hepatitis B and C virus infection, has increased in frequency and accounts for approximately 20% of HCC patients. [2] NAFLD exhibits a histological spectrum, ranging from “bland steatosis” to the more aggressive necro-inflammatory form, non-alcoholic steatohepatitis (NASH), which may lead to the accumulation of fibrosis, resulting in cirrhosis and HCC. The pathogenesis of NASH is still unclear and an effective treatment has not been established.

Autophagy is a lysosomal degradative pathway that promotes cell survival by supplying energy in times of stress. The involvement of autophagy in the pathogenesis of NAFLD was first suggested by the finding that autophagy mediates the breakdown of intracellular lipids in hepatocytes. [3] Growing evidence has supported the association of impaired autophagy with the development of NAFLD and progression of NASH. Moreover, a deficiency of autophagy is involved in tumorigenesis in the liver. [4]

Peretinoin (generic name; code, NIK-333), developed by Kowa Company (Aichi, Japan), is an oral acyclic retinoid with a vitamin A-like structure that targets the retinoid nuclear receptor. The oral administration of peretinoin significantly reduced the incidence of post-therapeutic HCC recurrence and improved the survival rates of patients in a clinical trial. [5, 6] A large-scale international clinical study is now planned to confirm the clinical efficacy of peretinoin. Although peretinoin treatment can suppress the growth of HCC-derived cell lines and inhibit experimental liver carcinogenesis of mouse or rat, [7, 8] the detailed mechanisms underlying its action have not been elucidated fully. Peretinoin has high binding affinity to cellular retinoic acid-binding protein and may interact with retinoic acid receptor-β and retinoid X receptor-α; [9] however, the precise molecular targets for preventing HCC recurrence have not been elucidated.

Previously, we showed that peretinoin significantly repressed the development of hepatic fibrosis and tumors by inhibiting the signaling pathways of fibrogenesis, angiogenesis, and Wnt/β-catenin using a mouse model in which PDGF-C is overexpressed. [10] In the present study, we evaluated the effects of peretinoin on autophagy, steatohepatitis, and HCC development using a diet-induced NASH mouse model.

## RESULTS

### Peretinoin improves steatosis, inflammation, and fibrosis in the liver of the atherogenic and high-fat diet-induced NASH mouse model

**Figure 1:**
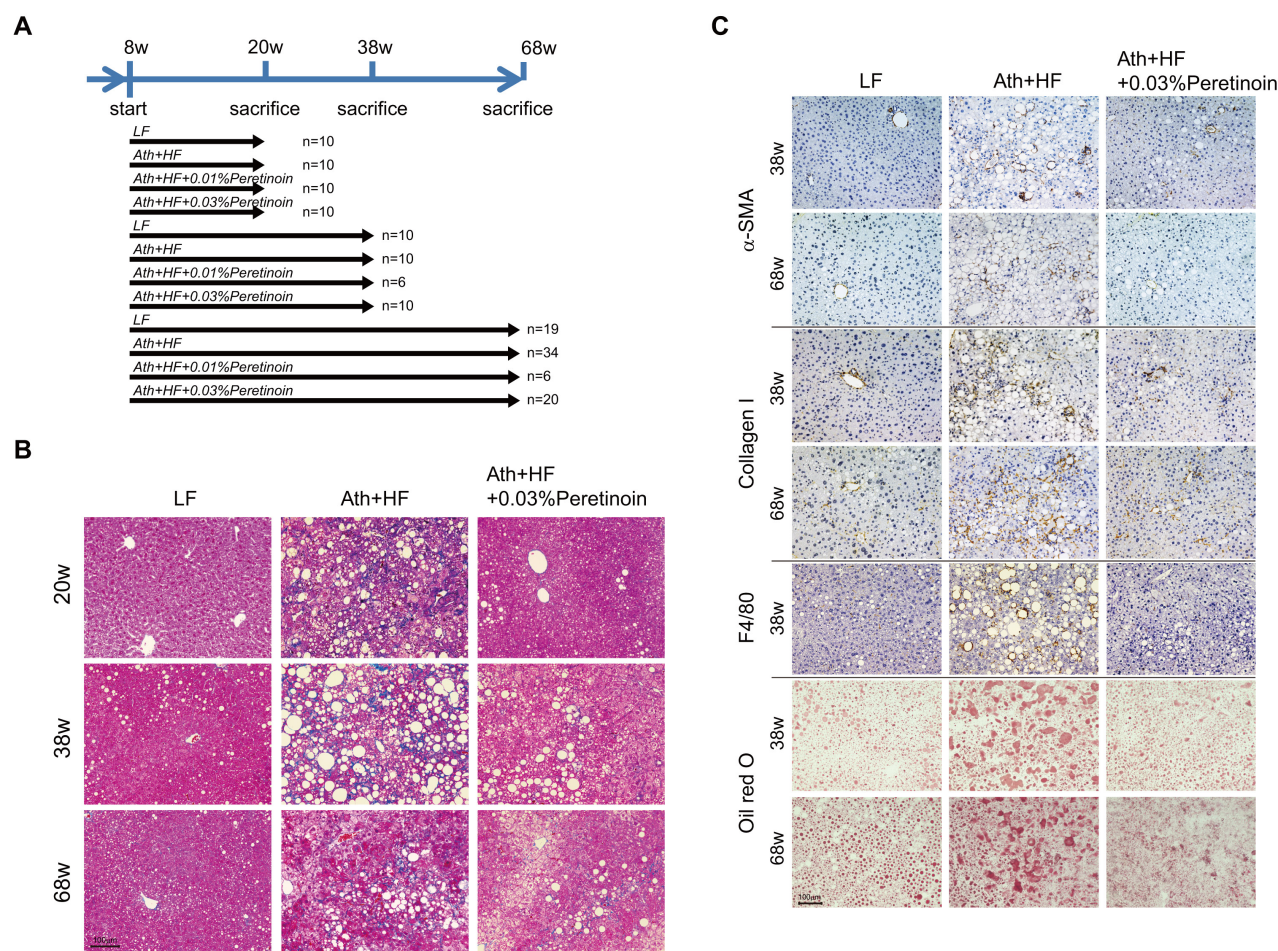
Histological improvement in the liver of Ath+HF diet mice by the addition of peretinoin **A**. Feeding schedule of the mice. After weaning, male C57BL/6J mice were divided randomly into 4 groups: (i) LF diet, (ii) Ath+HF diet, (iii) Ath+HF diet supplemented with 0.01% peretinoin, or (iv) with 0.03% peretinoin. **B**. Azan staining of mouse livers fed the LF, Ath+HF, or Ath+HF diet supplemented with 0.03% peretinoin at 20 weeks (20w), 38w, and 68w. **C**. Immunohistochemical staining for α-SMA, collagen 1, F4/80, and oil red O in the liver of mice fed the LF, Ath+HF, or Ath+HF diet supplemented with 0.03% peretinoin at 38w and 68w.

**Figure 2:**
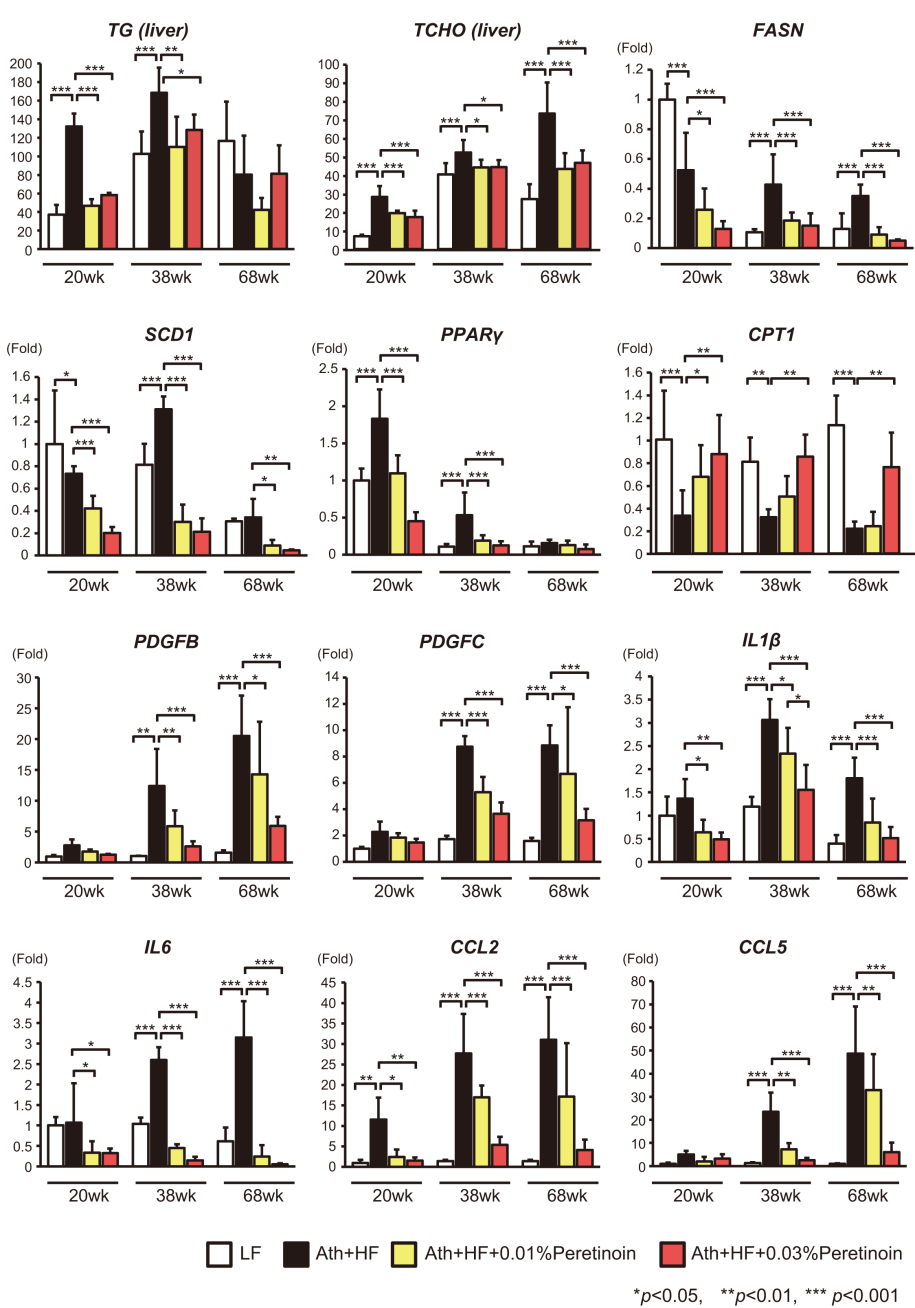
Effects of peretinoin on lipid metabolism, fibrosis, and inflammation Relative content of triglycerides (TG) and total cholesterol (TCHO), and the relative expression of FASN, SCD1, PPARγ, CPT1, PDGFB, PDGFC, IL1β, IL6, CCL2, and CCL5 mRNA in the liver of mice fed the LF, Ath+HF, or Ath+HF diet supplemented with 0.01% or 0.03% peretinoin at 20w, 38w, and 68w (*N* = 6).

We previously reported the atherogenic and high-fat (Ath+HF) diet-induced NASH mouse model (Materials and Methods). [11] These mice develop steatosis, inflammation, and fibrosis accompanied with cellular ballooning, a necessary histological feature defining human NASH, after 24 weeks. [12] In the present study, we continued to feed the mice until 68 weeks. The mice were sacrificed at 20, 38, and 68 weeks (Figure 1A), and liver histology and hepatic gene expression were analyzed (Figures 1 and 2 and Supplemental Figure 1). Azan staining and oil red O staining showed a substantial increase in the area of steatosis and fibrosis in the liver of Ath+HF diet mice compared with control low-fat (LF) diet mice. The inclusion of 0.03% peretinoin in the diet significantly improved liver histology (Figure 1A and 1C); 0.01% peretinoin also improved liver histology, but it was less effective than 0.03% peretinoin (data not shown). Immunohistochemical staining showed that the expression of α-SMA, collagen 1, and F4/80 were increased in Ath+HF diet mice and their expression was repressed by 0.03% peretinoin (Figure 1C).

Quantification of liver triglycerides and total cholesterol showed that triglycerides increased until 38 weeks, while total cholesterol increased until 68 weeks in Ath+HF diet mice (Figure 2). Peretinoin significantly reduced the levels of triglycerides and total cholesterol. Correlating with these findings, peretinoin significantly decreased the expression of FASN, SCD1, and PPARγ. Conversely, peretinoin restored the expression of CPT1, an important mitochondrial enzyme responsible for the β-oxidation of long-chain fatty acids. The expression of pro-fibrotic genes, such as PDGFB and PDGFC, and pro-inflammatory genes, such as interleukin (IL) 1β, IL6, CCL2, and CCL5, was increased in Ath+HF diet mice until 68 weeks, and their expression was repressed by peretinoin (Figure 2).

Hepatic gene expression profiling of differentially expressed genes (*p* < 0.005) in the liver of LF diet, Ath+HF diet, and Ath+HF diet with 0.03% peretinoin mice showed 3 gene clusters (clusters 1-3). Cluster 1 (489 genes) and cluster 2 (423 genes) contained genes that were up-regulated with disease progression, while the genes in cluster 3 (260 genes) were down-regulated with disease progression (Supplemental Figure 1). Cluster 1 included inflammation-related genes, while cluster 2 included fibrosis-related genes. The expression of these genes was repressed by peretinoin. Cluster 3 included metabolism-related genes, such as FXR-related, mitochondria-related, and autophagy-related genes. The expression of these genes was partially restored by peretinoin.

### Peretinoin prevents tumorigenesis in the liver of the NASH-HCC mouse model

**Figure 3:**
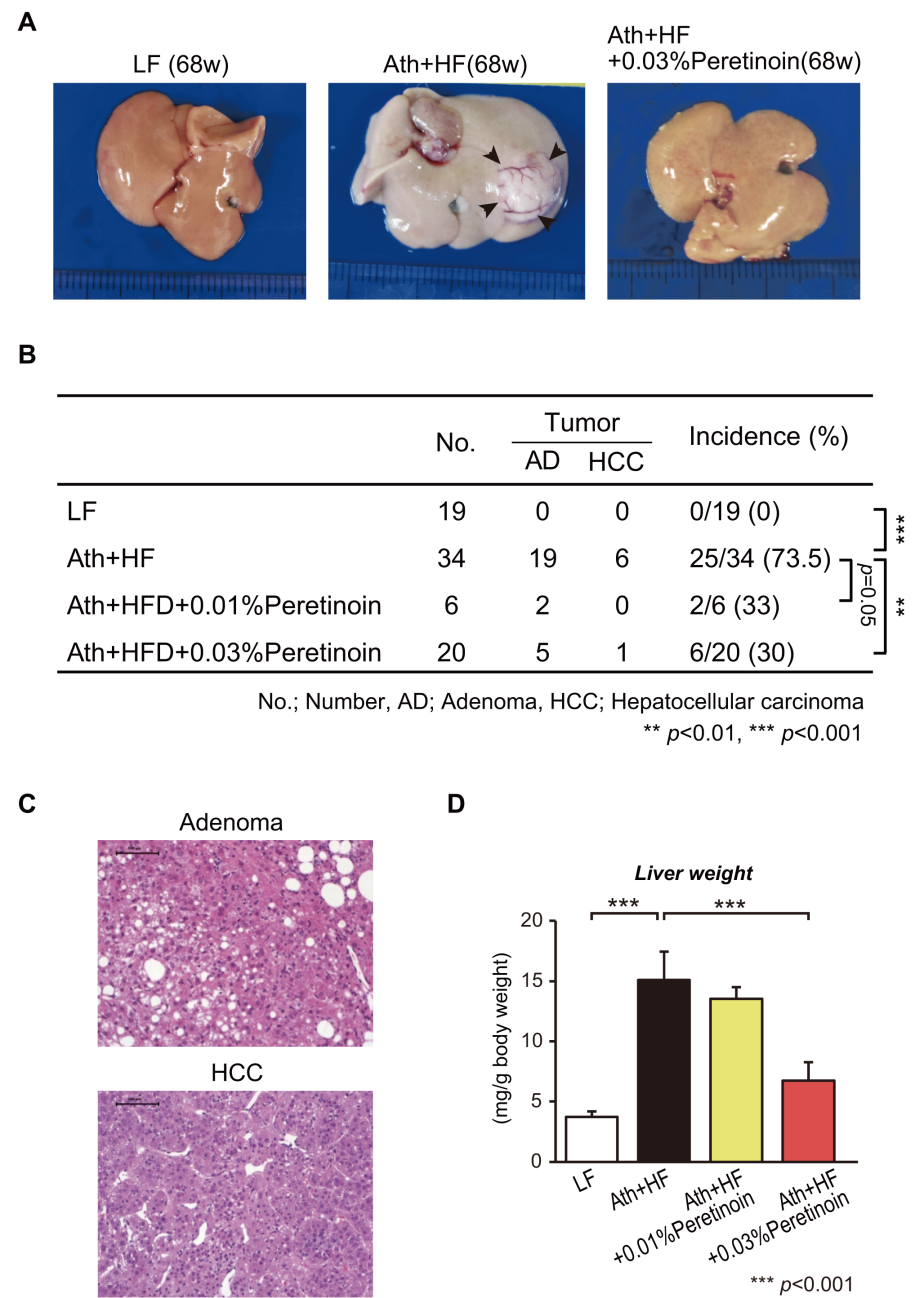
Effects of peretinoin on liver tumorigenesis in mice fed the Ath+HF diet **A**. Macroscopic findings and **B**. incidence of hepatic tumors (adenoma or HCC) in the liver of mice that were fed the LF, Ath+HF, or Ath+HF diet supplemented with 0.01% or 0.03% peretinoin at 68w. **C**. Hematoxylin and eosin staining of adenoma and HCC. **D**. Liver weights of mice fed the LF, Ath+HF, or Ath+HF diet supplemented with 0.01% or 0.03% peretinoin at 68w.

Ath+HF diet mice developed liver tumors at 68 weeks. The incidence of liver tumors was 73.5% (25/34), while no LF diet mice developed liver tumors (Figure 3A and 3B). Among the 25 mice that developed tumors, 19 (76%) were adenoma and 9 (24%) were HCC, and representative histology is shown in Figure 3C. The administration of 0.03% peretinoin significantly reduced the incidence of liver tumors (*p* < 0.01), and 0.01% peretinoin also reduced their incidence, but not significantly so (*p* = 0.05). Moreover, 0.03% peretinoin significantly reduced the liver weight of Ath+HF diet mice (Figure 3D).

The effect of peretinoin on tumorigenesis was confirmed by using another HCC mouse model, the STAM™ mouse. The STAM™ mouse is a streptozotocin-induced diabetic mouse model that was then fed the HF diet (Supplemental Figure 2A). [13] The STAM™ mouse represents NASH-like histology: steatosis, inflammation, and fibrosis at 8 weeks of age, and develops HCC at 100% by 20 weeks of age. Peretinoin was administrated to STAM™ mice from 6 weeks of age until 10 weeks of age (0.03% peretinoin, *n* = 4; 0.06% peretinoin, *n* = 3) or 22 weeks of age (0.03% peretinoin, *n* = 4). The effects of peretinoin on liver histology, gene expression, and tumorigenesis were compared with those from peretinoin-free STAM™ mice (Supplemental Figure 2A). Peretinoin improved hepatic steatosis and fibrosis (at 10 weeks of age; Supplemental Figure 2B) and reduced the incidence of hepatic tumors (at 22 weeks of age; Supplemental Figure 2C). Peretinoin significantly reduced the expression of CCL2, CCL5, IL1β, IL6, and TNFα in the liver of STAM™ mice (Supplemental Figure 2D).

### Peretinoin induces autophagy by increasing Atg5-Atg12-Atg16L1 pathway activation in the liver of the NASH-HCC mouse model

**Figure 4:**
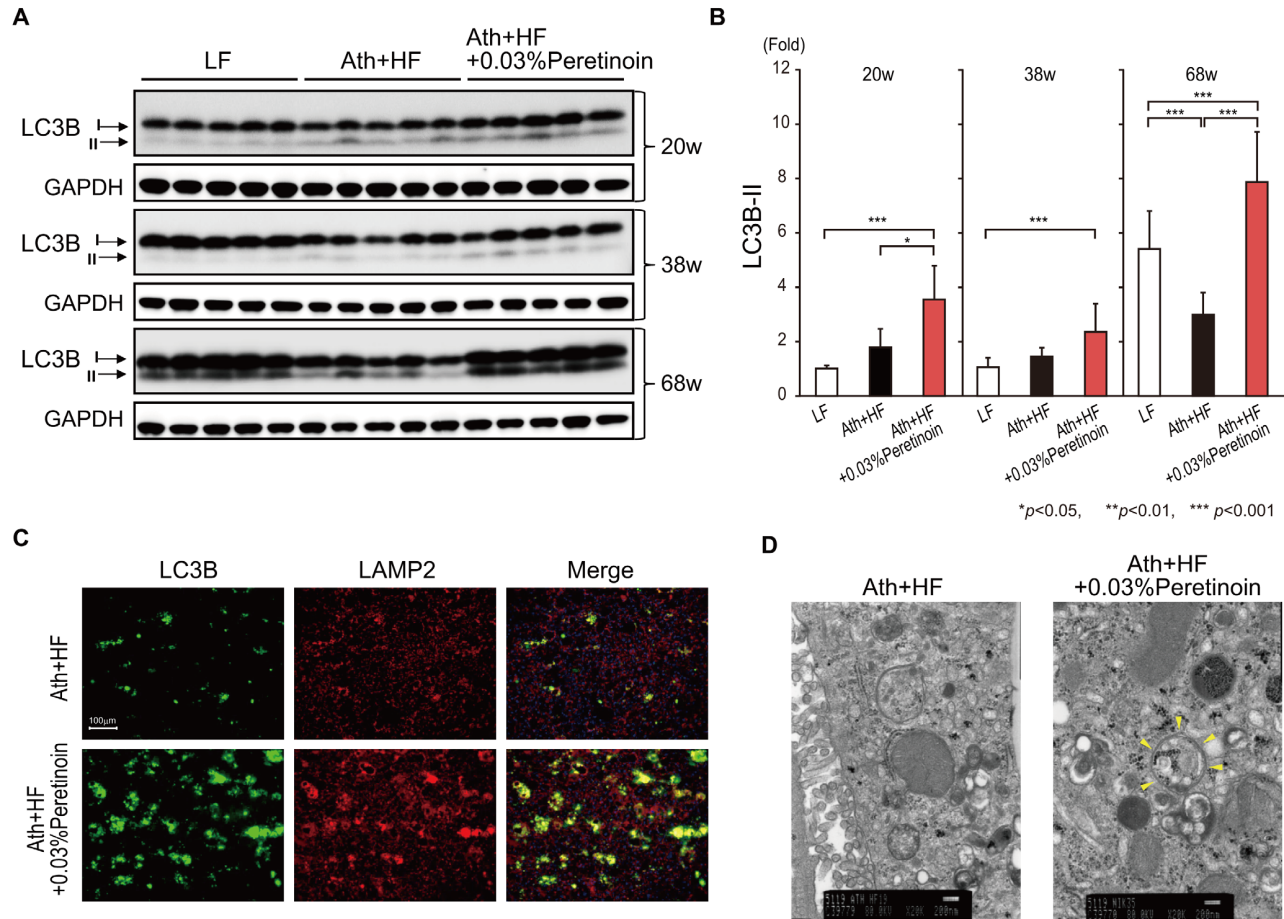
Peretinoin activates autophagy in the liver of mice fed the Ath+HF diet supplemented with 0.03% peretinoin **A**. Western blotting of LC3B-I and LC3B-II in the liver of mice that were fed the LF, Ath+HF, or Ath+HF diet supplemented with 0.03% peretinoin at 20w, 38w, and 68w. **B**. Densitometry semi-quantitation of the expression of LC3B-II by western blotting. **C**. Immunofluorescence staining of LC3B and LAMP2 in the liver of mice fed the Ath+HF or Ath+HF diet supplemented with 0.03% peretinoin at 68w. **D**. Electron microscopy findings in the liver of mice fed the Ath+HF or Ath+HF diet supplemented with 0.03% peretinoin at 68w. The arrowheads indicate an autophagosome.

To reveal the molecular mechanisms by which peretinoin prevented tumorigenesis in both NASH mouse models, we focused on the status of autophagy. We evaluated the expression of microtubule-associated protein light chain 3 (LC3B-II; phosphatidylethanolamine-conjugated LC3B), which is a critical component of double-membrane autophagosomes, in the liver of Ath+HF diet mice at 20, 38, and 68 weeks. At 20 and 38 weeks, no significant difference was observed in the expression of LC3B-II between the liver of LF diet and Ath+HF diet mice (Figure 4A and 4B). The administration of peretinoin weakly but significantly increased the expression of LC3B-II. At 68 weeks, the expression of LC3B-II in the liver of control LF diet mice increased as reported previously; [14] however, its expression did not increase in the liver of Ath+HF diet mice. The administration of peretinoin restored the expression of LC3B-II (Figure 4A and 4B). Immunofluorescence staining of liver sections showed the increased expression of LC3B and lysosome-associated membrane protein-2 (LAMP2; a lysosomal marker) by peretinoin and both markers co-localized, implying the fusion of autophagosomes and lysosomes undergoing autophagy (Figure 4C). Electron microscopy demonstrated that peretinoin increased autophagosome formation in the liver of Ath+HF diet mice (Figure 4D).

**Figure 5:**
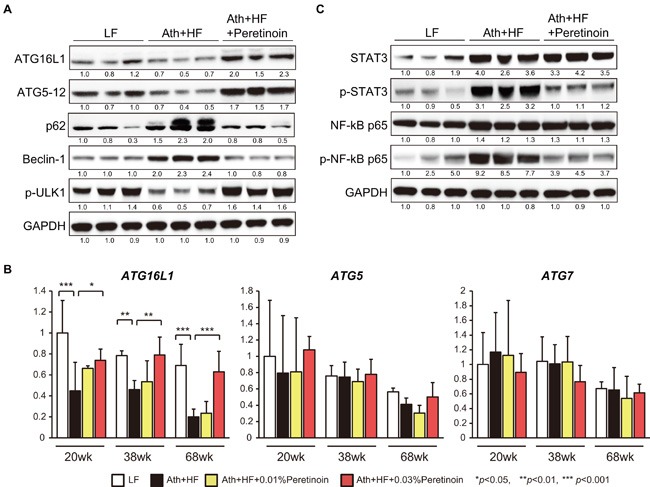
Atg16L1 expression is regulated by peretinoin at both the mRNA and protein level **A**. and **C**. Western blotting of Atg16L1, Atg5-12 complex, p62, Beclin-1, p-ULK1 STAT3, p-STAT3, NF-κB, and p-NF-κB in the liver of mice fed the LF, Ath+HF, or Ath+HF diet supplemented with 0.03% peretinoin at 68w. **B**. Relative expression of Atg16L1, Atg5, and Atg7 mRNA in the liver of mice fed the LF, Ath+HF, or Ath+HF diet supplemented with 0.03% peretinoin at 20w, 38w, and 68w.

To examine which autophagy pathway was activated by peretinoin; we examined 3 representative machineries engaged in the autophagy process: i) autophagy related (Atg) 5-Atg12-Atg16L1 complex, ii) ULK1 protein-kinase complex, and iii) VPS-Beclin1 class III PI3-kinase complex. Western blotting analysis showed that the expression of Atg16L1 and the Atg5-12 complex was decreased in the liver of Ath+HF diet mice and increased by peretinoin (Figure 5A). The expression of p62, a substrate of autophagy, was increased in the liver of Ath+HF diet mice and decreased by peretinoin. Conversely, the expression of Beclin1, an inducer of autophagy, was increased in the liver of Ath+HF diet mice and decreased by peretinoin. The expression of p-ULK1, a kinase of Beclin1, was decreased in the liver of Ath+HF diet mice and increased by peretinoin. Therefore, the Atg5-Atg12-Atg16L1 pathway, but not the Beclin1 and ULK1 pathways, could be involved in peretinoin-induced autophagy. Interestingly, Atg16L1 mRNA expression was decreased in the liver of Ath+HF diet mice and its expression was increased by peretinoin, while Atg5 and Atg7 mRNA expression was not changed (Figure 5B). We performed gene chip expression analysis of liver tissues obtained from 45 patients with NAFLD using an Affymetrix GeneChip (U133 Plus 2.0). The probe intensity of Atg16L1 was significantly decreased in the liver of NASH patients (NAFLD 3 and NAFLD 4) compared to fatty liver patients (NAFLD 1 and NAFLD 2) (Supplemental Figure 3), and no significant difference of expression was observed for Atg5 and Atg7 in the liver of NAFLD patients (data not shown). For inflammation status, the expression of phosphorylated (p)-signal transducer and activator of transcription 3 (STAT3) and p-nuclear factor kappa B (NF-κB) p65 was increased in the liver of Ath+HF diet mice and this expression was repressed by peretinoin (Figure 5C).

These findings were confirmed in STAM™ mice (Supplemental Figure 2). LC3B-II expression in the liver was increased by peretinoin (Supplemental Figure 2E). The expression of Atg16L1 and Atg5-12 complex was decreased in the liver of STAM™ mice (22 weeks compared to 10 weeks), and their expression was increased by peretinoin. Immunofluorescence staining confirmed the up-regulation of Atg16L1 by peretinoin in the liver of STAM™ mice (Supplemental Figure 2F). Conversely, the expression of p-STAT3 was increased in the liver of STAM™ mice (22 weeks compared to 10 weeks) and its expression was decreased by peretinoin (Supplemental Figure 2E). Thus, peretinoin enhanced autophagy by increasing Atg5-Atg12-Atg16L1 pathway activation and reduced steatosis, inflammation, and tumorigenesis in 2 different NASH-HCC mouse models.

### Peretinoin enhances autophagy in mouse primary hepatocytes

**Figure 6:**
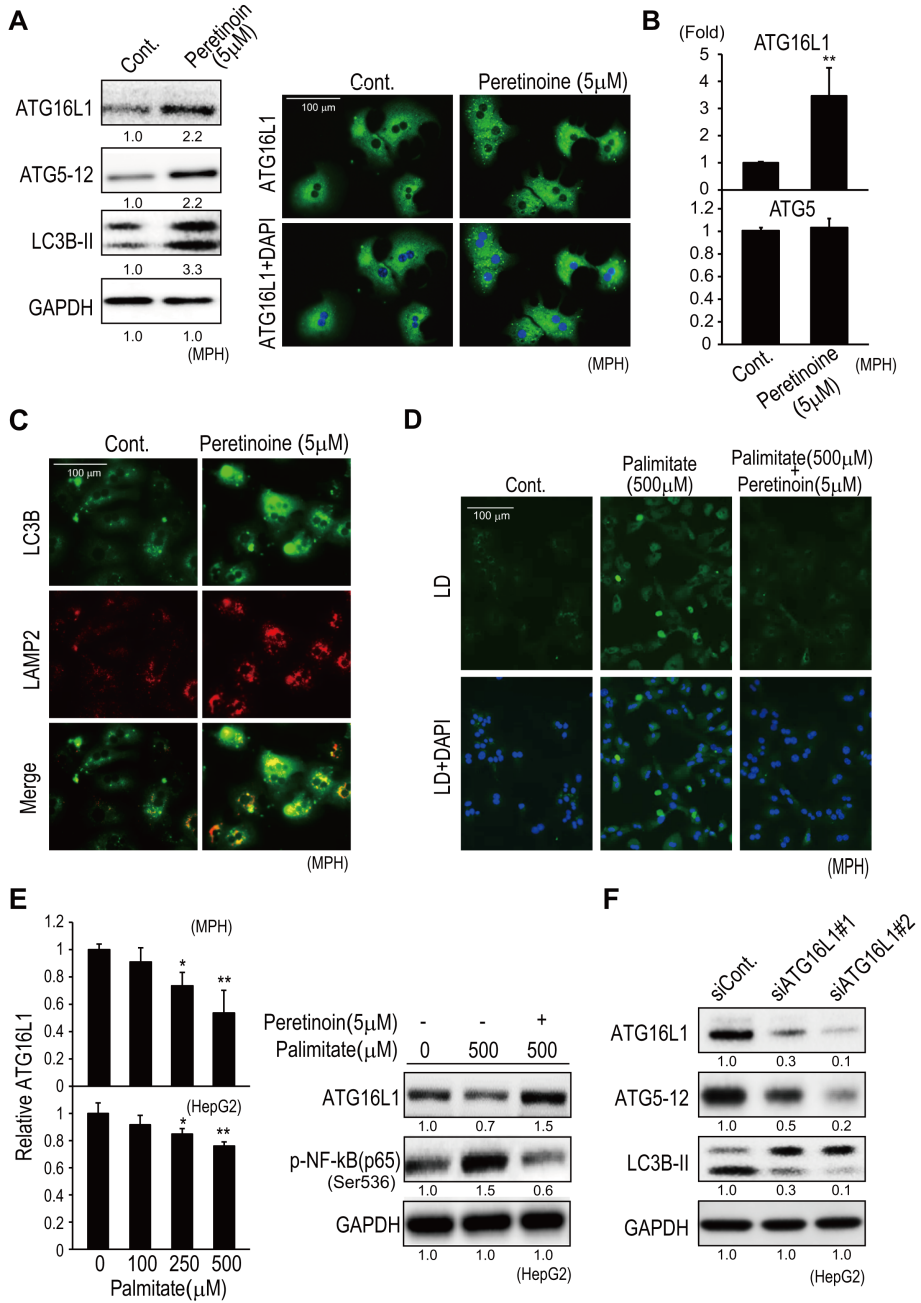
Peretinoin increases Atg16L1 expression and activates autophagy in MPH **A**. Increased expression of Atg16L1, Atg5-12 complex, and LC3B-II by peretinoin in MPH, determined by western blotting (left) and immunofluorescence staining (right). **B**. Relative expression of Atg16L1 and Atg5 mRNA in MPH treated with peretinoin. **C**. Immunofluorescence staining of LC3B and LAMP2 in MPH treated with peretinoin. **D**. Lipid droplets (LD) in MPH treated with palmitate in the presence or absence of peretinoin. **E**. Relative expression of Atg16L1 mRNA in MPH or HepG2 cells treated with palmitate (left). Western blotting for Atg16L1 and p-NF-κB in HepG2 cells treated with palmitate in the presence or absence of peretinoin (right). **F**. Knocking down Atg16L1 decreased the expression of the Atg5-12 complex and LC3B-II in HepG2 cells.

The effect of peretinoin on autophagy was confirmed in mouse primary hepatocytes (MPH) and the human HCC HepG2 cell line. Peretinoin increased the protein expression of Atg16L1, Atg5-12 complex, and LC3B-II in MPH (Figure 6A left). Immunofluorescence staining confirmed an increased number of Atg16L1-positive vesicles in peretinoin-treated MPH (Figure 6A right). Peretinoin increased Atg16L1 mRNA expression, but not Atg5 mRNA (Figure 6B), as observed in the Ath+HF diet mice (Figure 5). The expression of LC3B and LAMP2 was increased and co-localized in peretinoin-treated MPH (Figure 6C). An autophagy flux assay was performed by using chloroquine, which prevents the fusion of autophagosomes and lysosomes (Supplemental Figure 4). Chloroquine treatment further up-regulated the peretinoin-induced expression of LC3B-II, indicating that peretinoin increased autophagy flux.

Palmitate, a saturated fatty acid, has hepatocellular toxicity and initiates an inflammatory response to activate NF-κB signaling, which leads to the secretion of various cytokines and chemokines including IL6. [15] The addition of palmitate (500 μM) increased lipid droplet formation in MPH (Figure 6D), and peretinoin (5 μM) reduced lipid droplet formation in MPH (Figure 6D). Palmitate repressed the expression of Atg16L1 mRNA in MPH and HepG2 cells in a dose-dependent manner (Figure 6E left). The suppression of Atg16L1 and the induction of p-NF-κB by palmitate were completely inhibited by peretinoin (Figure 6E right). Thus, the palmitate-induced increase in lipid droplet formation, NF-κB activation, and suppression of Atg16L1 expression in hepatocytes were inhibited by peretinoin.

Interestingly, knocking down Atg16L1 by using specific small interfering RNAs decreased the expression of Atg16L1 together with the Atg5-12 complex and LC3B-II (Figure 6F). Therefore, Atg16L1 was indispensable for the formation of the Atg5-12 complex and LC3B-II that were needed following autophagy.

### Peretinoin inhibits IL6 signaling by increasing the expression of Atg16L1

**Figure 7:**
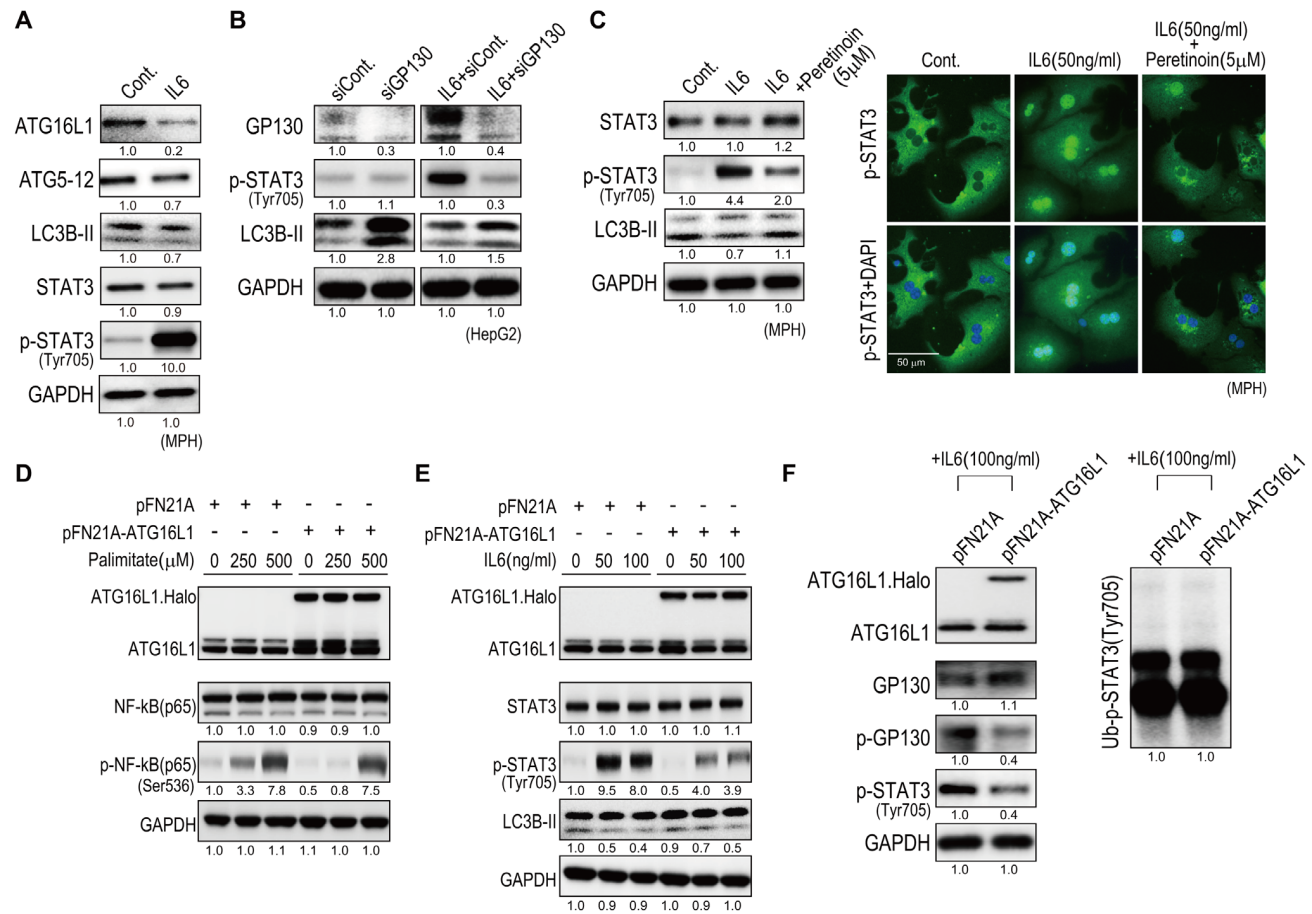
Peretinoin inhibits IL6 signaling by up-regulating Atg16L1 **A**. IL6 decreased the expression of Atg16L1, Atg5-12 complex, and LC3B-II in MPH. **B**. Knocking down Gp130 increased LC3B-II expression in HepG2 cells. **C**. Peretinoin restored the IL6-mediated suppression of LC3B-II (left) and inhibited the nuclear accumulation of p-STAT3 induced by IL6 in MPH. **D**. Overexpression of Atg16L1 and the suppression of palmitate increased p-NF-κB in HepG2 cells. **E**. Overexpression of Atg16L1 and the suppression of IL6 increased p-STAT3 in HepG2 cells. **F**. Western blotting of p-Gp130 using an anti-phosphotyrosine antibody following immunoprecipitation of Gp130 from HepG2 cells in which Atg16L1 was overexpressed (left). Ubiquitination of p-STAT3 using an anti-ubiquitin antibody (right).

We next examined the effect of peretinoin on IL6 signaling. The administration of IL6 to MPH decreased the expression of Atg16L1, Atg5-12 complex, and LC3B-II (Figure 7A). Knocking down Gp130, a signal transducer of IL6, increased the expression of LC3B-II in HepG2 cells (Figure 7B). Thus, IL6 signaling inhibited autophagy in hepatocytes. Interestingly, peretinoin suppressed the expression of IL6-induced p-STAT3 and restored the expression of LC3B-II (Figure 7C left). Immunofluorescence staining demonstrated that peretinoin suppressed the IL6-induced nuclear accumulation of p-STAT3 in MPH (Figure 7C right). Thus, peretinoin induced autophagy and suppressed the IL6-induced activation of p-STAT3.

As peretinoin increased the expression of Atg16L1 at both the mRNA and protein level, we examined the effect of Atg16L1 on NF-κB and IL6 signaling. The overexpression of Atg16L1 (Halo-tagged) in HepG2 cells decreased palmitate-induced NF-κB activation (Figure 7D). Similarly, the overexpression of Atg16L1 (Halo-tagged) in HepG2 cells decreased IL6-induced STAT3 activation (Figure 7E). Thus, the overexpression of Atg16L1 inhibited NF-κB and IL6 signaling; however, the increase of LC3B-II was subtle (Figure 7E). To reveal the effect of Atg16L1 on IL6 signaling in detail, changes in the phosphorylation of Gp130 by Atg16L1 were evaluated (Figure 7F left). Gp130 was immunoprecipitated and its phosphorylation status was evaluated using an anti-phosphotyrosine antibody. Atg16L1 overexpression decreased p-Gp130, which subsequently repressed the expression of p-STAT3 (Tyr705). Conversely, the ubiquitination status of p-STAT3 was not changed, indicating that the degradation of p-STAT3 was not affected (Figure 7F right). Thus, Atg16L1 could inhibit IL6 signaling through the de-phosphorylation of Gp130, possibly independently of autophagy.

### Peretinoin activates the Atg16L1 promoter by increasing the expression of the transcription factor CCAAT/enhancer binding protein alpha

**Figure 8:**
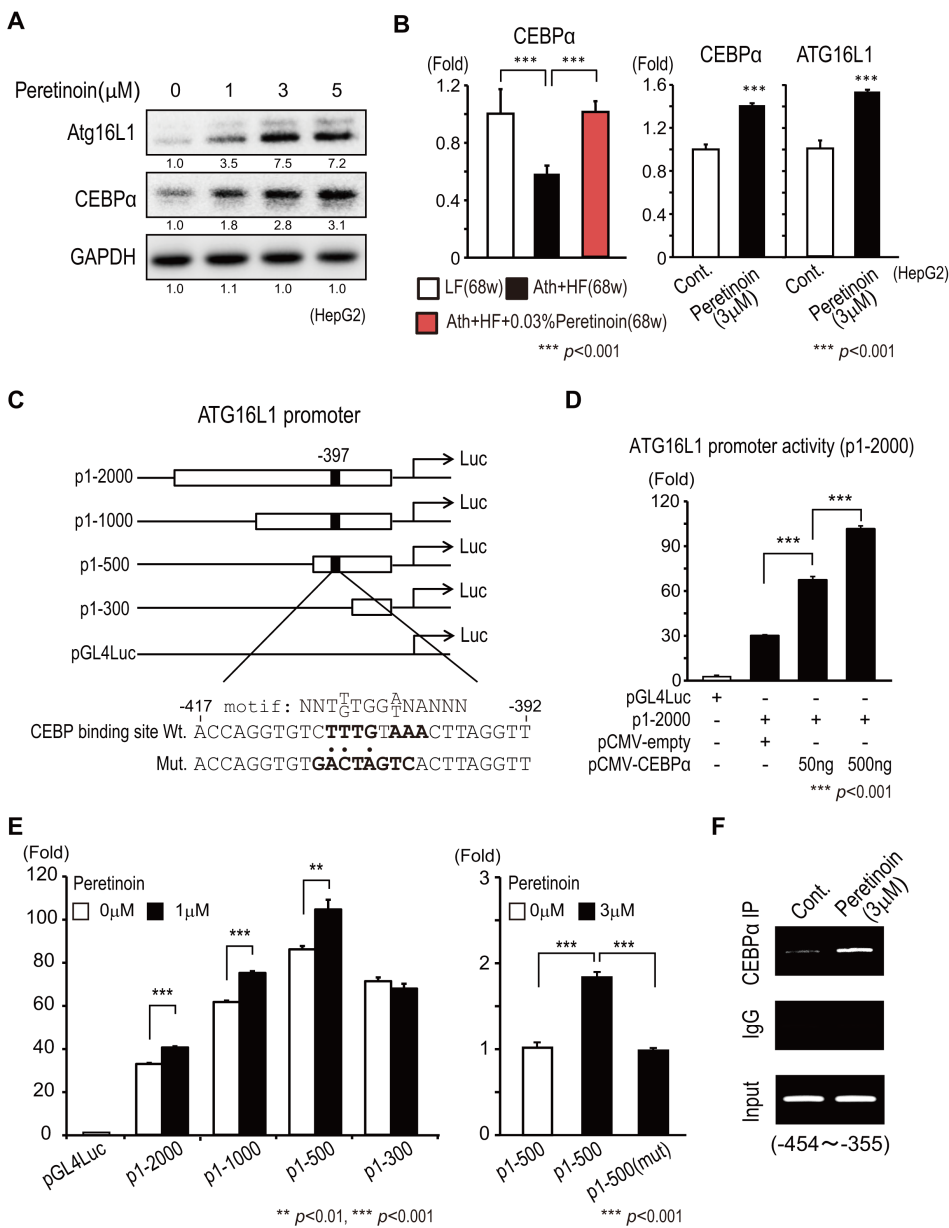
Transcriptional regulation of Atg16L1 by peretinoin through CEBPα **A**. Dose-dependent increase in CEBPα by peretinoin in HepG2 cells. **B**. Relative expression of CEBPα mRNA in the liver of mice fed the LF, Ath+HF, or Ath+HF diet supplemented with 0.03% peretinoin at 68w (*N* = 6) (left). Relative expression of CEBPα and Atg16L1 mRNA in HepG2 cells treated with peretinoin (right). **C**. Construction of Atg16L1 promoter assay constructs. P1-2000: including -2000 to 372 bp relative to the transcription initiation site of Atg16L1 fused to a firefly luciferase gene. P1-1000, P1-500, and P1-300: reporter constructs of serial deletions of the putative promoter region. Mutations were introduced to the CEBPα binding motif. **D**. Overexpression of CEBPα and promoter activity of Atg16L1. **E**. Effect of peretinoin on a series of Atg16L1 promoter assay constructs (left). Effect of peretinoin on p1-500 and p1-500 (mut), in which the putative CEBPα binding site was mutated (right). **F**. ChIP assay. HepG2 cells were treated with peretinoin and chromatin DNA was precipitated using an anti-CEBPα antibody.

As peretinoin increased the expression of Atg16L1 at the mRNA level, we examined the regulation of the Atg16L1 promoter by peretinoin using HepG2 cells. We focused on the hepatocyte differentiation transcription factor CCAAT/enhancer binding protein alpha (CEBPα), as we previously reported the up-regulation of CEBPα in the liver of patients who received peretinoin. [16] Peretinoin increased the protein expression of Atg16L1 and CEBPα in HepG2 cells in a dose-dependent manner (Figure 8A). Peretinoin significantly increased the mRNA level of CEBPα in the liver of Ath+HF diet mice and HepG2 cells (Figure 8B). We made reporter constructs of the serially truncated promoter region of Atg16L1 fused to a firefly luciferase gene (p1-2000, p1-1000, p1-500, and p1-300) (Figure 8C). p1-2000 showed Atg16L1 promoter activity that was 30-fold higher than background (pGL4Luc) (Figure 8D). CEBPα overexpression significantly increased Atg16L1 promoter activity (Figure 8D). To find the responsible region through which peretinoin acts on the promoter of Atg16L1, serially truncated promoter reporter gene constructs were examined. Peretinoin (1 µM) significantly increased the promoter activity of p1-2000, p1-1000, and p1-500, but not p1-300, indicating that a peretinoin-responsive region was located between -500 and -300 (Figure 8E left). We found a putative CEBP binding site located -397 bases upstream from the transcription start site of Atg16L1, and introduced mutations at this site using p1-500 (Figure 8C). Peretinoin (3 µM) significantly increased the promoter activity of p1-500, while it failed to increase the promoter activity of p1-500 (mut), in which the CEBP binding site was mutated (Figure 8E right). A chromatin immunoprecipitation (ChIP) assay showed that, in peretinoin-treated cells, immunoprecipitation of CEBPα could precipitate genomic DNA including the -397 CEBPα binding site (Figure 7F). These results indicated that peretinoin increased Atg16L1 promoter activity by increasing the expression of CEBPα.

## DISCUSSION

The lack of an appropriate experimental model for NASH-derived HCC is a barrier for developing effective therapies. We previously reported an Ath+HF diet mouse model that develops hepatic steatosis, inflammation, fibrosis, and insulin resistance, accompanied with cellular ballooning, a necessary histological feature defining human NASH. [12] In the present study, we continued to feed these mice until 68 weeks and observed the development of liver tumors at a high frequency (73.5%). We showed that peretinoin, an acyclic retinoid, significantly reduced the incidence of liver tumors to approximately 30% (Figure 3). Peretinoin is now under a phase III, placebo-controlled, multi-center, randomized, double-blind trial to evaluate recurrence in patients with complete cure of HCC in Japan and Asia. Analysis of gene expression in the liver of Ath+HF diet mice using a gene chip and quantitative real-time detection PCR showed that peretinoin markedly improved hepatic steatosis, inflammation, and fibrosis (Figure 2 and Supplemental Figure 1).

To reveal the underlying molecular mechanisms of peretinoin, we focused on the status of autophagy. Impaired autophagy is associated with lipid storage in hepatocytes and the development of NAFLD. [3, 17] Moreover, autophagy suppressed tumorigenesis in the liver of Atg5 or Atg7 knockout mice. [4] Interestingly, it was reported that all trans-retinoic acid promoted autophagy and induced the granulocytic differentiation of acute promyelocytic leukemia, although the precise mechanism has not been clarified. [18]

In the present study, we showed that autophagy was impaired in the liver of 2 NASH-HCC mouse models (Ath+HF diet mice and STAM™ mice). The suppression of autophagy in HF diet mice has been reported in several studies. [19–21] However, one of the differences of our model from previous reports was the long observation period (68 weeks in Ath+HF diet mice and 22 weeks in STAM™ mice) compared with the relative short observation period of previous studies (from 8 to 16 weeks). The impaired autophagy was restored by the administration of peretinoin (Figure 4 and Supplemental Figure 2), and this finding was more obvious in older mice, which would be associated with the increased autophagy observed in the liver of normal aged mice without tumors. [14]

Increased autophagy by peretinoin was clearly shown by the increased co-localized expression of the autophagy marker LC3B-II and lysosomal marker LAMP2, and autophagosome formation in hepatocytes by peretinoin (Figures 4C, 4D, and 6C). The increased formation of autophagosomes by peretinoin was not due to a delay of autophagy flux, which was increased by peretinoin (Supplemental Figure 4). Importantly, the expression of p62, a substrate of autophagy, was decreased by peretinoin (Figure 5A). A recent report showed that p62 is upregulated during pre-neoplasia and induces hepatocellular carcinogenesis by maintaining the survival of stressed HCC-initiating cells. [22]

Among the representative machineries engaged in the autophagy process, we found that the expression of the Atg5-Atg12 complex and Atg16L1 was decreased in Ath+HF diet mice (Figure 5A). Especially, the expression of Atg16L1 was transcriptionally regulated, while the expression of the Atg5-Atg12 complex was not (Figures 5A, 5B, and 6B). Atg16L1 reportedly forms a complex with the Atg5-Atg12 complex, and the resulting Atg5-Atg12-Atg16L1 complex plays essential roles in the conjugation of phosphatidylethanolamine to LC3B-I to generate LC3B-II, and these processes are required for subsequent autophagosome formation. [23] Although a recent report showed that Rubicon was increased in HF diet mice and inhibited autophagy, [20] the VPS-Beclin1 class III PI3-kinase pathway might not be involved in our model because the Rubicon-counteracting protein Beclin1 was increased in Ath+HF diet mice (Figure 5A). In the present study, the down-regulation of p-ULK1 and up-regulation of Beclin1 were contradictory to our observation that autophagy was suppressed in Ath+HF diet mice. Although we are unable to provide a clear explanation for this observation at present, a recent report showed that the Atg5 and ULK1 systems could promote autophagy independently. [24] Ulk1-mediated Atg5-independent autophagy mediates the elimination of mitochondria from embryonic reticulocytes. [25] In the condition of chronic liver disease, the reduced mTOR signaling in hepatocytes and increased growth factor expression in non-parenchymal cells might have differential effect on ULK1 and Atg5 autophagy mechanisms. Further study is necessary to clarify the functional differences of each autophagy process in advanced NASH.

Interestingly, knocking down Atg16L1 substantially repressed the expression of the Atg5-Atg12 complex (Figure 6F). Therefore, Atg16L1 might be involved in the stability of the Atg5-Atg12 complex in hepatocytes. Moreover, in the liver of patients with NAFLD, Atg16L1 expression was significantly decreased according to the progression of NAFLD (Supplemental Figure 3). Therefore, Atg16L1 appears to have an important role in the pathogenesis of NAFLD.

In this study, we showed that Atg16L1 expression was transcriptionally up-regulated by peretinoin (Figure 5B). We showed that peretinoin increased the promoter activity of Atg16L1 by increasing the expression of CEBPα, a well-known transcription factor for the differentiation of hepatocytes and adipocytes (Figure 8). We previously reported that CEBPα was induced in the liver of chronic hepatitis C patients who were treated with peretinoin. [16] We speculate that the induction of Atg16L1 by peretinoin through CEBPα might be RAR/RXR-independent, as the overexpression or suppression of RXRα did not affect Atg16L1 expression in HepG2 cells (data not shown).

The autophagy pathway and/or proteins also have a crucial role in the control of inflammatory signaling. [23] In the present study, IL6 expression was substantially up-regulated in the liver of Ath+HF diet mice and STAM™ mice, and this induction of IL6 was predominant in older mice (from 38 weeks to 68 weeks in the Ath+HF diet model and 22 weeks in STAM™ mice) (Figure 2 and Supplemental Figure 2D). However, the functional relevance of IL6 signaling on autophagy is controversial. [26] We showed that IL6 decreased the expression of Atg16L1, Atg5-Atg12 complex, and LC3B-II in MPH (Figure 7A). Therefore, IL6 signaling in NASH is considered to suppress autophagy and increase inflammation in the liver. We showed that peretinoin inhibited IL6 signaling, possibly by increasing Atg16L1 expression. Atg16L1 overexpression repressed the palmitate-induced activation of NF-κB and IL6-induced activation of STAT3 (Figure 7D and 7E). Furthermore, we revealed that Atg16L1 mediated the de-phosphorylation of Gp130, which subsequently repressed the expression of p-STAT3 (Tyr705). The direct interaction of Atg16L1 and Gp130 is now under investigation. Interestingly, Atg16L1 overexpression did not induce autophagy, as compared to the finding that knocking down Atg16L1 substantially repressed autophagy (Figures 6F and 7E). Therefore, Atg16L1 overexpression might have differential roles other than autophagy. A recent report showed that Atg16L1 is critical for the Nod-dependent regulation of cytokine responses, which was independent of autophagy. [27]

The Gp130-Jak-STAT3 pathway plays important roles in the inflammation process induced by IL6 family ligands such as IL11. IL11 is the dominant IL6 family cytokine during gastrointestinal tumorigenesis. [28] Further studies are needed to show the anti-tumor effect of Atg16L1 in the liver.

In conclusion, we showed that peretinoin prevents the progression of NASH and the development of HCC by activating the autophagy pathway through increasing the expression of the autophagy-essential and anti-inflammatory protein Atg16L1. These findings would be applicable and useful for the chemoprevention of NASH-derived HCC.

## MATERIALS AND METHODS

### Chemicals

Peretinoin ( [2E,4E,6E,10E]-3,7,11,15-tetramethyl-2,4,6,10,14-hexadecapentaenoic acid, C20H30O2, molecular weight 302.46 g/mol) was supplied by Kowa Company (Aichi, Japan).

### Animal studies

The generation and characterization of Ath+HF diet mice were performed as described previously. [11] Male C57BL/6J mice were maintained in a pathogen-free animal facility under a standard 12-h/12-h light/dark cycle. After weaning at week 8, male mice were divided randomly into 4 groups and each group was given one of the following diets for 12, 30, or 60 weeks: (i) LF basal diet, (ii) Ath+HF diet, or (iii) Ath+HF diet supplemented with 0.01% peretinoin or (iv) with 0.03% peretinoin. The LF diet contained 4.3% fat (cocoa butter and soybean oil), 19.2% protein (casein and L-cysteine), 66.4% carbohydrate (corn starch, maltodextrin, and sucrose), 4.3% mineral mixture, 0.9% vitamin mixture, 0.2% choline bitartrate, and 4.7% cellulose (Research Diets, New Brunswick, NJ). The Ath+HF diet contained 34.3% fat (cocoa butter and soybean oil), 25.8% protein (25.4% casein and 0.4% L-cysteine), 24.6% carbohydrate (corn starch, maltodextrin, and sucrose), 1.3% cholesterol, 0.5% cholic acid, 5.7% mineral mixture, 1.3% vitamin mixture, 0.2% choline bitartrate, and 6.3% cellulose (Research Diets, New Brunswick, NJ). The Ath+HF diet supplemented with 0.01% or 0.03% peretinoin contained 34.3% fat (cocoa butter and soybean oil), 25.8% protein (25.4% casein and 0.4% L-cysteine), 24.6% carbohydrate (corn starch, maltodextrin, and sucrose), 0.01% or 0.03% peretinoin, 1.3% cholesterol, 0.5% cholic acid, 5.7% mineral mixture, 1.3% vitamin mixture, 0.2% choline bitartrate, and 6.3% cellulose (Research Diets, New Brunswick, NJ). The mice were killed at week 20 and 38 to analyze the progression of hepatic steatosis and fibrosis or at week 68 to analyze the development of hepatic tumors. To generate STAM™ mice, [13] 2-day-old male C57BL/6N mice received a subcutaneous injection of 200 μg streptozotocin to reduce pancreatic function. Male STAM™ mice were purchased from Stelic Institute & Co. (Tokyo, Japan). Starting at 4 weeks of age, STAM™ mice were fed an HF diet (CLEA-Japan, Tokyo, Japan). Six-week-old male mice were divided randomly into 3 groups and each group was given one of the following diets for 4 or 16 weeks: (i) HF diet, (ii) HF diet supplemented with 0.01% peretinoin or (iii) with 0.03% peretinoin. The incidence of hepatic tumors and liver weight were evaluated. All animal experiments were carried out in accordance with the Guidelines for the Care and Use of Laboratory Animals of the Takara-machi Campus of Kanazawa University, Japan.

### Cell culture

The human hepatoblastoma HepG2 cell line was maintained in DMEM (Gibco, Grand Island, NY) supplemented with 10% fetal bovine serum (Gibco), 1% l-glutamine (Gibco), and 1% penicillin/streptomycin (Gibco) in a humidified atmosphere of 5% CO_2_ at 37°C. We seeded 1.0 × 10^5^ cells in each well of a 6-well plate. The culture medium was then replaced with serum-free medium containing peretinoin. After incubation for 24 h, the cells were harvested for analysis.

### Immunofluorescence staining

Frozen liver tissue sections and cultured cells were fixed in 4% paraformaldehyde for 15 min, rinsed in 0.1% Tween 20 in phosphate-buffered saline, and incubated in blocking buffer (DAKO, Tokyo, Japan). The primary and secondary antibodies were diluted in 1% bovine serum albumin/phosphate-buffered saline and incubated with the cells for 1 h at 37°C. The slides were then mounted using DAPI, and the cells were viewed using an image analysis system (BIOREVO BZ-9000; KEYENCE, Osaka, Japan). The following primary antibodies were used: rabbit anti-LC3B, rabbit anti-ATG16L1, rabbit anti-phospho STAT3 Tyr705 (Cell Signaling Technology, Inc., Danvers, MA), and mouse anti-LAMP2 (Abcam, Cambridge, MA). The slides were then incubated with Alexa Fluor 488 (goat anti-rabbit) and Alexa Fluor 594 (goat anti-mouse)-conjugated secondary antibodies (Invitrogen, Carlsbad, CA).

### Isolation and culture of MPH

MPH were isolated from 8-week-old male C57BL/6J mice as described previously. [29] Collagenase liver digestion was used to isolate primary hepatocytes. Primary hepatocytes were extracted from a liver cell suspension by a density gradient method using Percoll (Sigma-Aldrich, St. Louis, MO). All experiments were replicated at least twice. Freshly isolated primary hepatocytes suspended in culture medium were seeded in collagen-coated 6-well plates (IWAKI, Tokyo, Japan) and incubated at 37°C in a humidified atmosphere of 5% CO_2_ for 24 h. The culture medium was replaced with medium containing 50 ng/mL recombinant mouse IL6 (PeproTech, Rocky Hill, USA) with or without peretinoin. The cells were harvested for analysis after incubation for 24 h.

### Luciferase reporter assay

To generate reporter constructs for the luciferase assay, the promoter region of Atg16L1 containing a CEBPα binding site was inserted into the pGL4.10 vector (Promega Corporation, Madison, WI) using *Xho*I and *Kpn*I sites. Atg16L1 mutant (mut) promoter constructs were generated using a PrimeSTAR Mutagenesis Basal Kit (Takara Bio, Inc., Shiga, Japan). HepG2 cells were transfected with 500 ng of each reporter construct and 10 ng *Renilla* luciferase control plasmid (pRL-CMV; Promega) with/without 500 ng plasmid expressing CEBPα or empty control plasmid using Lipofectamine 2000 according to the manufacturer's protocol (Invitrogen). After incubation for 24 h, the cells were treated with 10% fetal bovine serum medium containing peretinoin. In the dual-luciferase assays, the cells were cultured for 24 h, and cell lysates were used to measure luciferase reporter gene expression using the dual luciferase reporter assay system (Promega).

### ChIP assay

A ChIP assay was carried out using a ChIP IT Express Enzymatic Kit (Active Motif, Carlsbad, CA) according to the manufacturer's instructions. HepG2 cells were treated with peretinoin for 24 h before being fixed and homogenized. Following centrifugation, the supernatant was used for chromatin samples. Chromatin samples were incubated with protein G-coated magnetic beads and a ChIP-grade anti-CEBPα antibody (Cell Signaling) overnight at 4°C. Following washing and elution, a reaction solution was used as the template for PCR. The resulting precipitated DNA samples were analyzed by PCR. The PCR products were resolved by electrophoresis on a 3% agarose gel and visualized with ethidium bromide staining.

### Statistical analysis

The results are expressed as the mean ± standard deviation. Significance was tested by one-way analysis of variance with Bonferroni's method and differences were considered statistically significant at *p* < 0.05.

### SUPPLEMENTARY MATERIALS FIGURES AND TABLES



## Abbreviations

NASH: Non-Alcoholic Steatohepatitis
HCC: Hepatocellular Carcinoma
Ath+HF: Atherogenic and High-Fat
LC3B: Microtubule-Associated Protein Light Chain 3
LAMP2: Lysosome-Associated Membrane Protein-2
Atg16L1: Autophagy Related 16 Like 1
Atg5: Autophagy Related 5
Atg12: Autophagy Related 12
STAT3: Signal Transducer and Activator of Transcription 3
CEBPα: CCAAT/Enhancer Binding Protein Alpha
ChIP: Chromatin Immunoprecipitation
IL6: Interleukin 6
NF-κB: Nuclear Factor Kappa B
NAFLD: Non-Alcoholic Fatty Liver Disease
LF: Low-Fat
MPH: Mouse Primary Hepatocytes
Riuta Takabatake: mouse and cellular experiments and construction of expression vectors
Masao Honda: study design, interpretation of data, and drafting the manuscript
Kai Takegoshi: acquisition of data of mouse experiments
Taro Yamashita: acquisition of data of clinical samples
Mikiko Nakamura: acquisition of data of gene chip and microRNA array
Takayoshi Shirasaki: acquisition of data of cellular experiments
Yoshio Sakai: acquisition of data of clinical samples
Tetsuro Shimakami: acquisition of data of clinical samples
Naoto Nagata: acquisition of data of cellular experiments
Toshinari Takamura: acquisition of data of clinical samples
Takuji Tanaka: acquisition of data of mouse liver histology
Shuichi Kaneko: study concept and design.
