# Supplementary material for: Peretinoin, an acyclic retinoid, suppresses steatohepatitis and tumorigenesis by activating autophagy in mice fed an atherogenic high-fat diet
Source: Oncotarget. 2017 May 23;8(25):39978–93. doi: 10.18632/oncotarget.18116 (PMC5522259; doi:10.18632/oncotarget.18116)
Supplement: Supplementary file 1 [file oncotarget-08-39978-s001.pdf]

Peretinoin, an acyclic retinoid, suppresses steatohepatitis and tumorigenesis by activating autophagy in mice fed an atherogenic high-fat diet

Supplementary Material

Patients

Liver tissue samples were obtained from 45 patients with NAFLD. The activity and stage of liver histology were classified into 4 types (type 1 = 9, type 2 = 8, type 3 = 17, and type 4 = 11) according to Matteoni's classification.(1)

Histopathology and immunohistochemical staining

Mouse liver tissues were fixed in 10% formalin and stained with hematoxylin and eosin. Liver neoplasms (HCC and liver cell adenoma) were diagnosed according to previously described criteria.(2) Hepatic fibrosis was evaluated by Azan staining. The percent of fibrous areas was calculated microscopically using an image analysis system (BIOREVO BZ-9000; KEYENCE, Osaka, Japan). Immunohistochemical staining was conducted by an immunoperoxidase technique with an Envision Kit (DAKO, Tokyo, Japan). The following primary antibodies were used: rabbit polyclonal collagen 1 (1:100 dilution; Abcam, Cambridge, MA), mouse monoclonal smooth muscle actin (1:100 dilution; DAKO), and rat monoclonal F4/80[CI:A3-1] (1: 200 dilution; Abcam).

Lipid droplet accumulation

To measure lipid drop accumulation *in vivo*, frozen liver tissue sections were fixed in 10% formalin for 1 h, washed with 60% isopropanol, and stained with oil red-O (Sigma-Aldrich, St. Louis, MO) solution for 10 min. The tissues sections were then washed repeatedly with water, photographed, and de-stained in 100% isopropanol for 15 min. To assess the effect of lipid droplet accumulation *in vitro*, cultured cells fixed in 4% paraformaldehyde were stained with BODIPY 493/503 Lipid Probes (Invitrogen, Carlsbad, CA). Nuclei were labeled with DAPI (Vector Laboratories, Burlingame, CA). The tissue sections and cultured cells were viewed using an image analysis system (BIOREVO BZ-9000; KEYENCE).

Blood sampling and analysis

At 20, 38, and 68 weeks, blood samples were obtained from the inferior vena cava of sacrificed mice following a 12-h fast. Enzymatic assays for total cholesterol and triglycerides were performed with kits purchased from Wako Pure Chemical Industries (Osaka, Japan).

Quantitative real-time detection PCR

Total RNA was isolated from frozen liver tissue samples and cultured cells using a High Pure RNA Tissue Kit (Roche Applied Science, Indianapolis, IN) according to the manufacturer's protocol. cDNA was synthesized from 100 ng total RNA using a High-capacity cDNA Reverse Transcription Kit (Applied Biosystems, Carlsbad, CA) and then mixed with the TaqMan Universal Master Mix (Applied Biosystems) and each TaqMan probe. The following TaqMan probes were used: FASN, SCD1, PPARγ, CPT1, PDGFB, PDGFC, IL1β, IL6, CCL2, CCL5, CEBPα, TNFα, Atg16L1, Atg5, and Atg7 (Applied Biosystems). Relative expression levels were calculated after normalization to glyceraldehyde-3-phosphate dehydrogenase (GAPDH) or 18S rRNA.

Western blotting and immunoprecipitation

Western blotting was conducted as described previously.(2) Whole-cell lysates from mouse liver were prepared and lysed using the CelLytic MT cell lysis reagent (Sigma-Aldrich, St. Louis, MO) containing Complete Mini EDTA-free Protease Inhibitor cocktail tablets (Roche). Cultured cells were washed in phosphate-buffered saline and lysed in RIPA buffer containing Complete Protease Inhibitor Cocktail and PhosSTOP (Roche Applied Science). The membranes were blocked in Block Ace (DS Pharma Biomedical Co., Ltd., Osaka, Japan). Primary antibodies used were LC-3B (1:1,000 dilution), Atg16L1 (1:1,000 dilution), Beclin-1 (1:1,000 dilution), p-ULK1 (1:1,000 dilution), p62 (1:1,000 dilution), STAT3 (1:1,000 dilution), p-STAT3 (Tyr705; 1:1,000 dilution), NFkB p65 (1:1,000 dilution), p-NFkB p65 (Ser536; 1:1,000 dilution), CEBPα (1:1,000 dilution), and GP130 (1:1,000 dilution; all Cell Signaling Technology); Atg5 (1:500 dilution; Novus Biologicals, Littleton, CO); and GAPDH (1:1,000 dilution; Santa Cruz Biotechnology, Inc., Santa Cruz, CA). Immunoprecipitation of GP130 was carried out using a Dynabeads protein G immunoprecipitation kit (Invitrogen) according to the manufacturer's instructions. Immunoprecipitated GP130 was then applied to western blotting to detect phosphorylated GP130 using an anti-phosphotyrosine antibody (Abcam).

Amino acid free medium (zero medium)

Zero medium was prepared by mixing 5.81 g nutrition-free DMEM (Nacalai Tesque, Kyoto, Japan), 1.85 g $NaHCO_3$, 1 g glucose, and 0.5 mL of 1 M (mol/L) sodium pyruvate in 500 mL Mili-Q water, then sterilizing with a 0.22-μm filter (Millipore, Billerica, MA).

Gene expression profiling

Gene expression profiling of mouse liver was performed using a GeneChip Mouse Gene 1.0 ST Array (Affymetrix, Santa Clara, CA).(3) Liver tissue was obtained from mice fed the basal, Ath+HF, or Ath+HF diet containing 0.03% peretinoin for 30 or 60 weeks. Pathway analysis was conducted using MetaCore (Thomson Reuters, New York, NY). Functional ontology enrichment analysis was conducted to compare the Gene Ontology process distribution of the differentially expressed genes.

DNA transfection

The expression vector for Atg16L1 and GP130 with the Halo tag was purchased from KAZUSA DNA Research Institute (Chiba, Japan). Putative Stat3 activating domain site-mutations of the GP130 vector were generated using a PrimeSTAR Mutagenesis Basal Kit (Takara Bio, Inc., Shiga, Japan), according to the manufacturer's instructions.

HepG2 cells were seeded in each well of a 6-well plate; 1 μg plasmid DNA with 2 μL Lipofectamine 2000 (Invitrogen) were added to each well. After 48 h, the culture medium was replaced with serum-free or amino acid-free medium containing 50 ng/mL recombinant human IL6 (PeproTech). After a 6-h incubation, the cells were harvested for analysis.

RNA interference

Small interfering RNAs (siRNAs) specific to Atg16L1 and GP130 and Low DC control siRNA were obtained from Invitrogen. Transfection was performed with Lipofectamine RNAiMAX (Invitrogen) according to the manufacturer's instructions.

REFERENCES

1. Matteoni CA, Younossi ZM, Gramlich T, Boparai N, Liu YC, McCullough AJ. Nonalcoholic fatty liver disease: a spectrum of clinical and pathological severity. Gastroenterology. 1999;116:1413-1419.

2. Okada H, Honda M, Campbell JS, Sakai Y, Yamashita T, Takebuchi Y, Hada K, Shirasaki T, Takabatake R, Nakamura M, Sunagozaka H, Tanaka T, Fausto N, Kaneko S. Acyclic retinoid targets platelet-derived growth factor signaling in the prevention of hepatic fibrosis and hepatocellular carcinoma development. Cancer Res. 2012;72:4459-4471.

3. Okada H, Honda M, Campbell JS, Takegoshi K, Sakai Y, Yamashita T, Shirasaki T, Takabatake R, Nakamura M, Tanaka T, Kaneko S. Inhibition of microRNA-214 ameliorates hepatic fibrosis and tumor incidence in platelet-derived growth factor C transgenic mice. Cancer Sci. 2015;106:1143-1152.

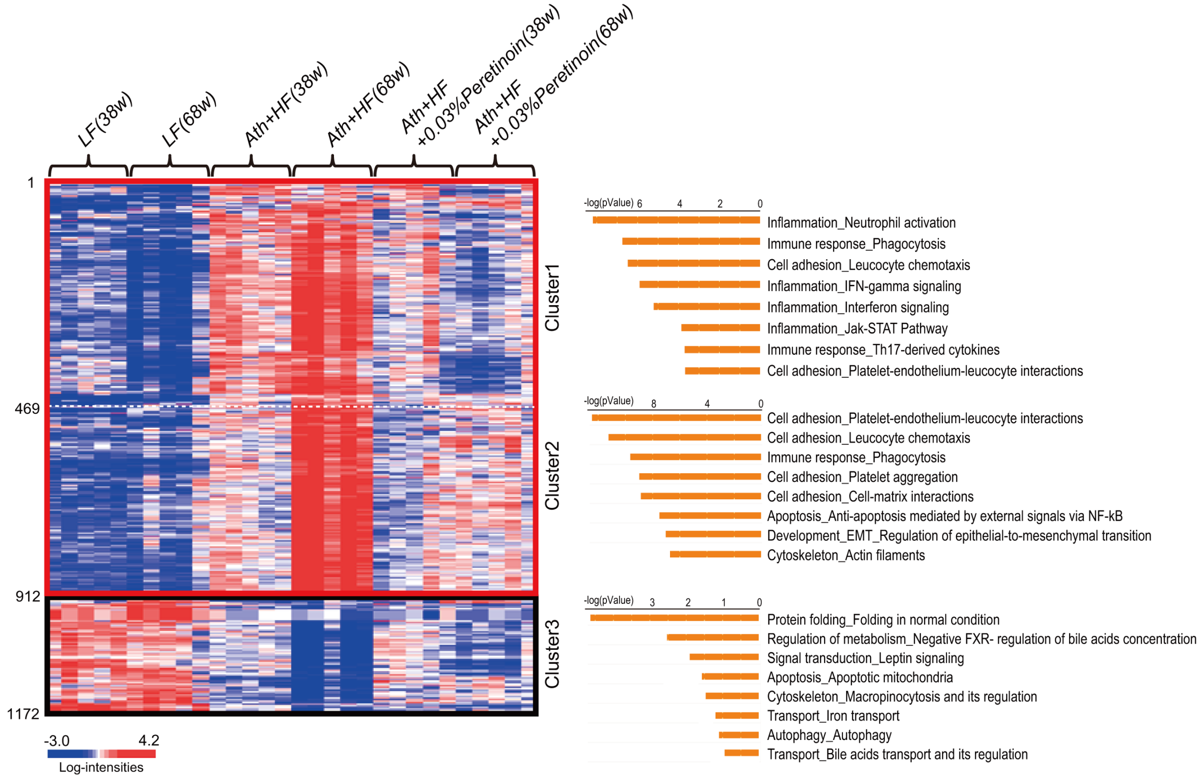

Supplemental Figure 1. One-way hierarchical analysis of 1172 genes expressed differentially between the livers of mice fed the LF, Ath+HF, or Ath+HF diet supplemented with 0.03% peretinoin at 38w and 68w (fold > 1.5, p < 0.005). Gene categories of each cluster deduced using MetaCore™ are shown on the right.

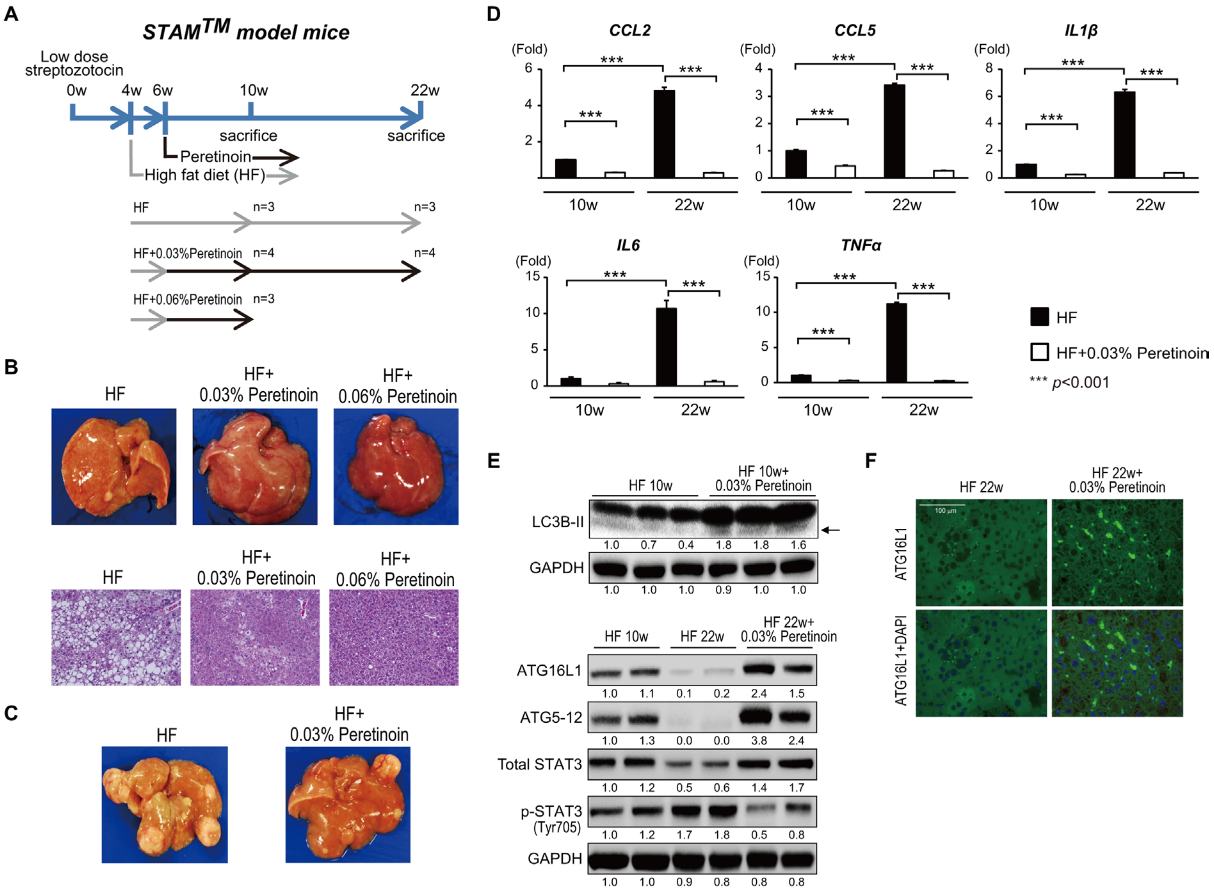

Supplemental Figure 2. Effect of peretinoin on STAM^{TM} mice. (A) Feeding schedule of the mice. After weaning, STAM^{TM} mice were divided randomly into 3 groups: (i) HF diet, (ii) HF diet supplemented with 0.03% peretinoin, and (iii) HF diet supplemented with 0.06% peretinoin. Liver histology and tumorigenesis were analyzed at 10w and 22w. (B) Macroscopic findings (upper) and hematoxylin and eosin staining (lower) of background liver of STAM^{TM} mice fed the HF diet, HF diet supplemented with 0.03% peretinoin, or HF diet supplemented with 0.06% peretinoin at 10w. (C) Macroscopic liver findings of STAM^{TM} mice fed the HF diet or HF diet supplemented with 0.03% peretinoin at 22w. (D) Relative expression of CCL2, CCL5, IL1β, IL6, and TNFα mRNA in the liver of STAM^{TM} mice fed the HF diet or HF diet supplemented with 0.03% peretinoin at 10w and 22w. (E) Western blotting of LC3B-II, Atg16L1, the Atg5-12 complex, STAT3, and p-STAT3 in the liver of STAM^{TM} mice fed the HF diet or HF diet supplemented with 0.03% peretinoin at 10w and 22w. (F) Immunofluorescence staining of Atg16L1 in the liver of STAM^{TM} mice fed the HF diet or HF diet supplemented with 0.03% peretinoin at 22w.

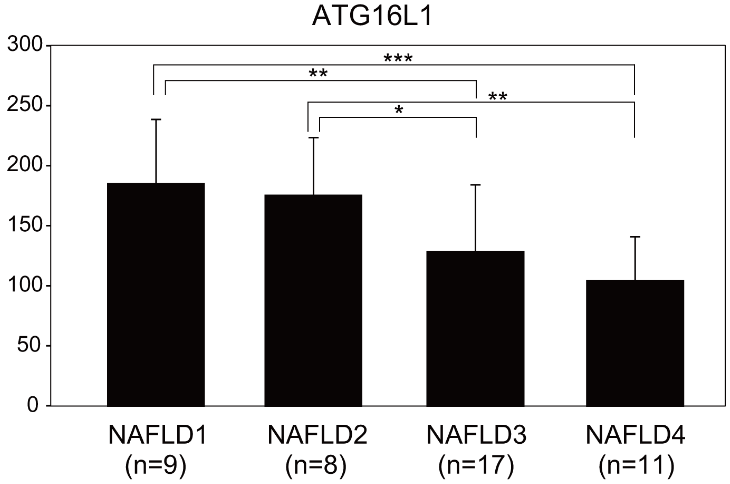

Supplemental Figure 3. Probe intensities of Atg16L1 derived from an Affymetrix GeneChip using liver samples from 45 patients with NAFLD.

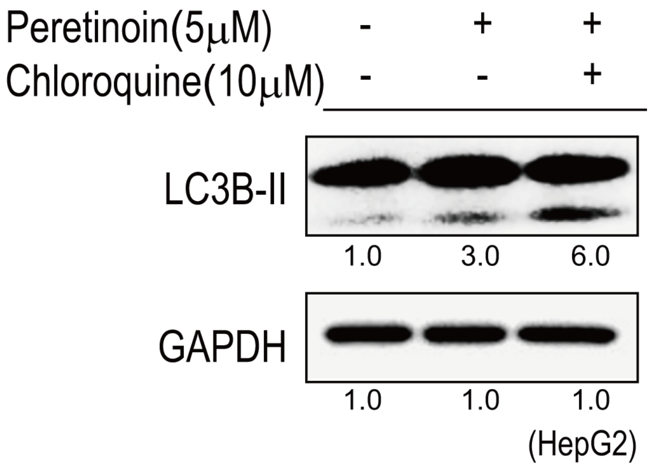

Supplemental Figure 4. Autophagy flux analysis using chloroquine. Peretinoin induced the expression of LC3B-II, and chloroquine, an inhibitor of the fusion of autophagosomes and lysosomes, further increased LC3B-II expression.
